# Restoring Satiety After GLP-1/GIP Pharmacotherapy: Metabolic Stability, Diet Quality, and the Gut Microbiota

**DOI:** 10.3390/ijms27114658

**Published:** 2026-05-22

**Authors:** Lidia Lasik, Natalia Ukleja-Sokołowska

**Affiliations:** 1 Independent Researcher, Poradnia Żywieniowa Akaja, 89-100 Nakło nad Notecią, Poland; lidialasik@wp.pl; 2 Department and Clinic of Allergology, Clinical Immunology and Internal Diseases, Ludwik Rydygier Collegium Medicum in Bydgoszcz, Nicolaus Copernicus University in Toruń, 87-100 Toruń, Poland

**Keywords:** obesity, GLP-1 receptor agonists, weight regain, satiety, metabolic adaptation, gut microbiota

## Abstract

GLP-1 receptor agonists and dual GLP-1/GIP agonists have significantly transformed the treatment of obesity, enabling clinically meaningful weight reduction and improvements in cardiometabolic parameters. However, clinical trial data indicate that cessation of therapy is associated with biologically driven weight regain and a partial loss of metabolic benefits. This phenomenon underscores the chronic nature of obesity and the limited durability of effects achieved through pharmacotherapy alone. Nevertheless, structured clinical frameworks describing how to maintain satiety and metabolic stability after GLP-1/GIP dose reduction or discontinuation remain limited. The aim of this narrative review is to discuss the mechanisms underlying weight regain following dose reduction or discontinuation of GLP-1/GIP pharmacotherapy and to present strategies supporting long-term metabolic stabilisation. Weight regain is driven in part by persistent metabolic adaptations, including a reduction in resting energy expenditure (adaptive thermogenesis), alterations in the hunger–satiety axis (increased ghrelin, reduced leptin signalling), and potentially incomplete restoration of adipose tissue and liver-related metabolic function, although direct evidence in this specific setting remains limited. Weight loss is often accompanied by a reduction in fat-free mass, which further lowers energy expenditure and increases susceptibility to a positive energy balance after treatment cessation. It remains unclear whether pharmacological suppression of appetite results in sustained normalisation of endogenous satiety regulation after treatment cessation, and its effects on gut microbiota function remain uncertain. In clinical practice, key priorities include preserving muscle mass (adequate protein intake, resistance training), maintaining dietary nutrient density, stabilising postprandial glycaemia, and ensuring sufficient intake of fermentable fibre to support short-chain fatty acid production and gut–brain signalling. GLP-1/GIP pharmacotherapy should be viewed as a component of an integrated model of obesity treatment. We propose that long-term weight stabilisation may require a transition from pharmacologically induced satiety to satiety supported by diet quality, preserved fat-free mass, and metabolic stability. Further research is needed to define optimal post-treatment strategies and to identify patients in whom therapy can be safely reduced or discontinued. This transition should be regarded as a conceptual framework and forward-looking hypothesis requiring validation in prospective studies.

## 1. Introduction

The introduction of glucagon-like peptide-1 (GLP-1) receptor agonists and dual GLP-1/GIP agonists has substantially altered the therapeutic approach to obesity. These agents demonstrate high efficacy in reducing body weight by suppressing appetite, delaying gastric emptying, modulating central appetite regulation, and improving glycaemic control [1,2]. Randomised trials, such as STEP 1 and STEP 4, have achieved weight reductions previously difficult to attain through lifestyle intervention alone [1,3].

At the same time, accumulating evidence indicates that discontinuation or dose reduction is associated with significant weight regain and a partial loss of previously achieved metabolic benefits. In the STEP 1 extension trial, following semaglutide withdrawal, approximately two-thirds of the lost weight was regained within one year [4]. Similar conclusions have been drawn from recent systematic reviews addressing the discontinuation of anti-obesity pharmacotherapy [5,6]. This phenomenon highlights the chronic nature of obesity and suggests that the efficacy of incretin-based therapy in weight management depends not only on continued pharmacological exposure but also on the quality of concurrent non-pharmacological strategies.

Obesity is a systemic disease characterised by chronic low-grade inflammation, impaired endocrine function of adipose tissue, and compromised metabolic flexibility [7]. Dysregulation of the gut–liver–brain axis and impaired insulin signalling contribute to altered mechanisms governing hunger and satiety [8]. While pharmacological modulation of GLP-1/GIP receptors effectively influences central appetite control, it may not lead to sustained normalisation of underlying metabolic disturbances, and this has not been directly demonstrated in post-discontinuation settings, nor to full restoration of metabolic flexibility.

Key adaptations following weight reduction include changes in energy expenditure and hormonal signalling (e.g., leptin, ghrelin), which favour weight regain [9]. Additionally, weight reduction is accompanied by a partial loss of fat-free mass, potentially lowering resting metabolic rate and complicating long-term maintenance of therapeutic effects [10,11].

During GLP-1/GIP pharmacotherapy, a significant reduction in energy intake is observed. While therapeutically desirable, this effect may lead to insufficient protein and micronutrient intake, particularly in the absence of targeted nutritional education [11,12]. Adequate protein intake and strategies to preserve muscle mass (e.g., resistance training) are considered key elements in minimising undesirable fat-free mass loss during incretin-based therapy [11].

Increasing attention is also being paid to the role of the gut microbiota in regulating satiety and metabolic stability. Obesity is associated with reduced microbial diversity and impaired production of short-chain fatty acids (SCFAs), which influence gut–brain signalling, insulin sensitivity, and energy metabolism [13,14].

Pharmacological appetite suppression alone may not be sufficient to restore functional gut microbiota or increase endogenous SCFA production; however, this has not been directly demonstrated in GLP-1/GIP post-treatment settings, and evidence in this context remains limited.

This may be relevant to weight regain after treatment cessation. These interpretations are partly based on indirect evidence from related metabolic models and should be interpreted with caution.

Despite the growing body of evidence on the efficacy of GLP-1/GIP agonists, current clinical guidelines focus primarily on indications and safety, whereas post-treatment strategies—encompassing diet quality, muscle preservation, and support of the gut–liver–brain axis—remain relatively underdescribed in current guidance [6,15]. However, structured clinical models describing the transition from pharmacologically induced satiety to diet- and metabolism-driven satiety remain insufficiently defined.

**Figure 1:**
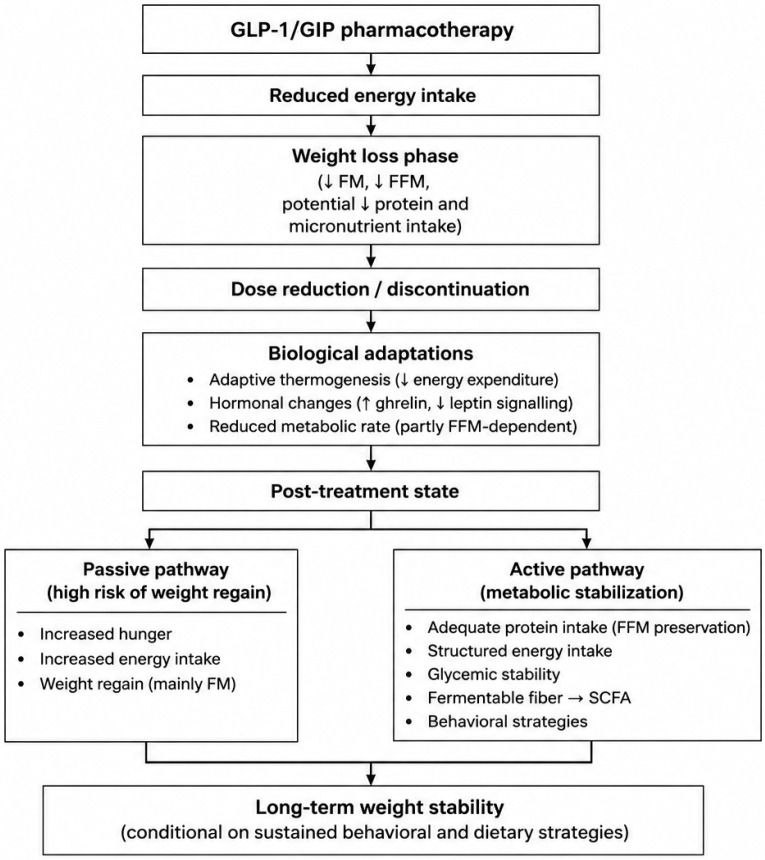
Conceptual model of post-treatment weight regulation following GLP-1/GIP pharmacotherapy. The model illustrates the transition from pharmacologically induced satiety to post-treatment physiological adaptations and highlights two potential pathways: passive weight regain and active metabolic stabilisation through diet-driven interventions. Arrows indicate the proposed progression from pharmacological appetite suppression to post-treatment metabolic outcomes and long-term weight stability pathways.

To integrate current evidence and conceptualise the transition from pharmacological to diet-driven satiety, we propose a conceptual model of post-treatment weight regulation (Figure 1).

### 1.1. Methods/Search Strategy

This narrative review was conducted using a structured literature search approach to identify clinically relevant evidence on weight regain, metabolic adaptations, and nutritional factors associated with GLP-1 receptor agonists and dual GLP-1/GIP agonists.

Electronic databases including PubMed/MEDLINE and Google Scholar were searched for studies published between 2005 and 2026. The search strategy combined terms related to obesity pharmacotherapy and metabolic regulation, including: GLP-1 receptor agonists, GIP, tirzepatide, semaglutide, weight regain, adaptive thermogenesis, fat-free mass, protein intake, gut microbiota, short-chain fatty acids, satiety, and metabolic adaptation.

Priority was given to randomised controlled trials, systematic reviews, meta-analyses, and high-quality clinical studies, particularly those from the STEP and SURMOUNT trial programmes. Mechanistic studies and selected preclinical or translational research were included where necessary to support biological plausibility, with explicit recognition of their inferential nature.

Given the narrative design of the review, formal inclusion and exclusion criteria were not predefined; however, emphasis was placed on studies with clear clinical relevance to obesity management and post-treatment weight stability. Particular attention was given to evidence addressing the period following dose reduction or discontinuation of incretin-based pharmacotherapy. The final literature search was conducted in February 2026. Reference lists of key articles were additionally screened to identify relevant publications.

### 1.2. Should GLP-1/GIP Pharmacotherapy Be Considered Chronic Treatment?

A key, unresolved question in contemporary obesity management is whether treatment with GLP-1 receptor agonists and dual GLP-1/GIP agonists should be regarded as a time-limited intervention or as chronic therapy, analogous to the treatment of hypertension or type 2 diabetes. This issue is particularly pertinent given evidence that the efficacy of these agents in weight reduction is closely dependent on continued use, whereas discontinuation is associated with biologically driven weight regain [3,4].

Randomised trials within the STEP programme have demonstrated that pharmacological enhancement in incretin signalling enables substantial weight loss during active treatment. In STEP 1, once-weekly semaglutide 2.4 mg resulted in clinically significant weight reduction and improvements in cardiometabolic parameters compared with placebo [1]. The STEP 5 trial showed that continued treatment maintained weight reduction for at least 104 weeks, indicating the durability of the therapeutic effect under sustained drug exposure [16].

However, in the STEP 1 extension, following semaglutide withdrawal, participants regained approximately two-thirds of their initial weight loss over one year, accompanied by partial reversal of metabolic improvements [4]. Similar findings emerged from STEP 4, where treatment discontinuation after an induction phase led to significant weight regain despite continued lifestyle advice, whereas ongoing treatment allowed for stabilisation or further reduction in body weight [3].

A comparable pattern of dependence on continued treatment has been demonstrated for the dual GLP-1/GIP agonist. In the SURMOUNT-4 trial, discontinuation of tirzepatide following an initial weight-loss phase resulted in weight regain, whereas continued treatment enabled maintenance and further reduction [17]. These data consistently indicate that, after discontinuation, weight regain is common; however, whether incretin-based pharmacotherapy leads to sustained normalisation of the biological mechanisms regulating energy balance remains insufficiently demonstrated. During active treatment, these agents appear to function primarily as modulators of disrupted energy homeostasis.

This interpretation aligns with current understanding of the metabolic adaptations accompanying weight loss. Following weight reduction, resting energy expenditure declines beyond what would be predicted from weight loss alone, a phenomenon termed adaptive thermogenesis [9]. Concurrently, alterations occur in the hormonal signalling that regulates appetite, including increased ghrelin levels and persistently reduced leptin concentrations, which favour increased hunger [5]. These mechanisms may persist long after weight loss and are thought to contribute to the biological drive toward weight regain. The duration of these adaptations is highly variable and may depend on the magnitude of weight loss, rate of regain, and individual metabolic phenotype. In this context, discontinuing GLP-1/GIP agonists may unmask compensatory mechanisms that were previously pharmacologically attenuated.

Recent systematic reviews confirm that weight regain after pharmacotherapy cessation is not limited to individual STEP trials. A systematic review on discontinuation of GLP-1 receptor agonists found that treatment cessation was associated with significant weight gain, irrespective of concurrent lifestyle interventions [5]. Similarly, a meta-analysis encompassing various classes of anti-obesity medications showed that stopping treatment led to rapid weight regain and partial loss of metabolic benefits, an effect also observed for incretin-based therapies [6]. At a population level, this suggests that pharmacotherapy alone may be insufficient to sustain control of the biological drivers of obesity after discontinuation.

In light of this, there is a growing consensus that obesity should be treated as a chronic disease requiring long-term pharmacotherapy in selected patients. This approach is partially reflected in international documents. The World Health Organization, in its guideline on the use of GLP-1 receptor agonists for obesity treatment, emphasises the need to embed pharmacotherapy within a long-term treatment plan, acknowledging the chronic nature of the disease [15].

Further dimension to this discussion is provided by data on hard endpoints. The SELECT trial demonstrated that semaglutide reduces the risk of cardiovascular events in patients with overweight or obesity and established cardiovascular disease, without type 2 diabetes [18]. This suggests that the benefits of therapy may extend beyond weight reduction alone, further supporting long-term use in patients at high cardiovascular risk.

At the same time, available data do not support the conclusion that GLP-1/GIP pharmacotherapy should be chronic for all patients. A more individualised approach appears appropriate, considering metabolic phenotype, degree of obesity, presence of comorbidities, rate of weight regain, and feasibility of implementing concurrent nutritional and behavioural strategies. It is important to emphasise that considering therapy potentially long-term does not imply its use in isolation. Failure to preserve fat-free mass, inadequate protein intake, or lack of strategies to stabilise energy balance may limit the possibility of safe discontinuation and promote weight regain [9,10].

Thus, in clinical practice, the question should not be solely “does the drug work?” but rather “in whom, in which metabolic context, and for how long does its use confer the greatest clinical benefit with an acceptable safety profile?” This perspective allows for reconciliation of the chronicity of obesity with the need for rational, individualised pharmacotherapy. This supports the need to conceptualise post-treatment care as a transition from pharmacologically supported appetite control to diet- and metabolism-supported satiety regulation.

## 2. Pathophysiology of Obesity Relevant to Weight Regain

### 2.1. Adipose Tissue as an Endocrine Organ

In obesity, adipose tissue is not merely a passive energy store but functions as an active endocrine organ whose dysfunction significantly influences energy balance regulation and susceptibility to weight regain following reduction. In obesity, leptin resistance, reduced adiponectin concentrations, and increased production of pro-inflammatory cytokines such as TNF-α and IL-6 are observed, contributing to persistent low-grade systemic inflammation [7,19].

Chronic inflammation within adipose tissue is associated with macrophage infiltration and activation of NF-κB and JNK pathways, which are associated with impaired insulin and leptin signalling [19]. Central leptin resistance, despite high circulating leptin levels, limits the hypothalamus’s ability to appropriately suppress appetite and increase energy expenditure [20]. Consequently, after weight loss, a relative deficit in satiety signalling may emerge in relation to reduced adipose tissue mass, promoting compensatory increases in energy intake.

Concurrently, reduced adiponectin concentrations may impair metabolic flexibility and the ability to adapt to changes in energy supply. Weight loss does not always fully normalise the adipokine profile, and persistent endocrine dysfunction of adipose tissue may contribute to renewed energy storage after the cessation of a weight-loss intervention [7,19].

### 2.2. Metabolic Liver Dysfunction

An important element in the pathophysiology of weight regain is metabolic liver dysfunction, particularly in the context of metabolic dysfunction-associated steatotic liver disease (MASLD) and hepatic insulin resistance [8]. These disturbances may contribute to dysregulation of gluconeogenesis, increased endogenous glucose production, and impaired metabolic flexibility, potentially influencing central processing of hunger and satiety signals.

The liver is a key component of the gut–liver–brain axis, integrating hormonal (GLP-1, PYY), metabolic (glucose, fatty acids), and neural (vagus nerve) signals [21]. Under conditions of hepatic insulin resistance, this axis may become dysregulated, and feedback between the availability of energy substrates and central signalling may be impaired.

Although steatosis and hepatic insulin resistance often improve with clinically meaningful weight loss, more advanced components of MASLD, particularly fibrosis and macrophage-driven inflammation, may be more resistant to full resolution. Therefore, any proposed contribution of residual liver dysfunction to appetite regulation after GLP-1/GIP discontinuation should be interpreted cautiously.

The specific contribution of residual hepatic insulin resistance to appetite dysregulation after GLP-1/GIP discontinuation has not yet been demonstrated in clinical studies.

### 2.3. Disturbances in Hunger–Satiety Signalling

Individuals with obesity exhibit complex disturbances in the hormonal axis regulating appetite, involving ghrelin, peptide YY (PYY), cholecystokinin, and endogenous GLP-1 [20,22]. During weight loss, ghrelin concentrations increase, and anorexigenic signals are relatively weakened, which may contribute to heightened hunger and increased energy intake.

Studies have shown that hormonal changes after weight loss may persist for at least 12 months and are consistent with a biological “defence” of higher body weight [22,23]. The persistence of these adaptations is variable across studies and may depend on the magnitude of weight loss, rate of regain, and individual metabolic phenotype.

Pharmacological modulation of these mechanisms with GLP-1/GIP agonists effectively suppresses appetite during treatment, but after discontinuation, it remains unclear whether endogenous hunger–satiety signalling undergoes sustained normalisation, although weight regain is frequently observed.

Furthermore, chronic inflammation and insulin resistance may affect hypothalamic neurotransmission, weakening the postprandial satiety response [19,20]. Consequently, after pharmacotherapy ends, individuals may exhibit increased sensitivity to hunger stimuli alongside attenuated satiety signalling, which may contribute to susceptibility to weight regain.

### 2.4. Loss of Fat-Free Mass and Energetic Adaptations

An additional, often underestimated, contributor to weight regain is the loss of fat-free mass during weight reduction, particularly when energy and protein intakes are low. Weight loss is typically associated with a loss of approximately one-quarter of fat-free mass [10], which is associated with a reduction in basal metabolic rate.

Adaptive thermogenesis may persist long after weight loss, which may result in total energy expenditure remaining lower than predicted based on current body weight [9,24,25]. Combined with heightened hunger and impaired satiety signalling, this may create a metabolic environment conducive to weight regain.

These mechanisms may help explain why weight regain is not solely a consequence of eating behaviours but reflects persistent biological adaptations that extend beyond the period of active treatment [5,9,25].

## 3. Impact of GLP-1/GIP Pharmacotherapy on Nutritional Status

Pharmacotherapy with GLP-1 receptor agonists and dual GLP-1/GIP agonists is associated with a significant reduction in energy intake, reflecting pharmacological appetite suppression, delayed gastric emptying, and enhanced postprandial satiety signals [1,2]. In clinical trials, reduced spontaneous energy consumption constitutes one of the primary mechanisms underlying observed weight loss. In clinical practice, this phenomenon may be accompanied by periods of marked energy restriction or irregular eating patterns, which are not always planned or monitored for dietary quality.

Although this mechanism promotes effective weight loss, increasing evidence suggests that sustained very low energy intake may be associated with reduced dietary quality and a potential risk of suboptimal nutrient intake, especially in the absence of structured dietary support [9,26,27]. Unlike controlled dietary interventions, pharmacologically induced spontaneous hypophagia may result in a disproportionate restriction of specific food groups, including sources of protein and fats.

### 3.1. Micronutrients and Qualitative Undernutrition

Reduced fat intake may affect the bioavailability of fat-soluble vitamins (A, D, E, K), whose absorption depends on the presence of lipids in the intestinal lumen [28]. Additionally, reduced total food volume and limited intake of animal-derived products may be associated with reduced intake of vitamin B12, iron, and zinc. Although direct data evaluating the frequency of micronutrient deficiencies during GLP-1/GIP therapy remain limited, similar mechanisms have been described in other models of prolonged energy restriction [29]. These comparisons should be interpreted cautiously, as they are inferential rather than based on direct micronutrient deficiency data from GLP-1/GIP cohorts.

It is important to note that subclinical reductions in dietary quality or nutrient adequacy may occur without overt symptoms in the initial phase of treatment. Potential consequences may include impaired muscle function, concentration difficulties, or reduced exercise tolerance, which may become more apparent after pharmacotherapy cessation or during dose reduction, when pharmacological appetite suppression is reduced or withdrawn.

### 3.2. Protein Intake and Muscle Mass

Protein intake is of particular importance in the context of incretin-based therapy. Reduced appetite and early satiety may contribute to a decrease in total protein consumption, which may be particularly relevant in older populations where the risk of sarcopenia is elevated. Earlier work by Wolfe and Miller suggested that standard protein intake recommendations (0.8 g/kg body weight) may be insufficient under conditions of weight loss and pronounced metabolic adaptations [12]. More recent recommendations in the context of incretin-based therapy provide a more directly applicable framework for protein intake and muscle preservation [11,12,30,31,32].

Recent recommendations for minimising muscle loss during incretin-based therapy emphasise the importance of protein intake in the range of at least 1.0–1.2 g/kg body weight, and in selected cases even higher, combined with resistance training [11]. Data from body composition analyses in the STEP and SURMOUNT programmes suggest that a proportion of weight loss constitutes fat-free mass reduction [10,16,17]. Although this proportion falls within the range observed in other weight-loss interventions, its clinical significance may increase with long-term treatment.

### 3.3. Fat-Free Mass Loss and Metabolic Consequences

Loss of fat-free mass is associated with a reduction in resting energy expenditure and total energy expenditure [10,24,25]. These adaptations may persist after weight loss cessation and may increase susceptibility to a positive energy balance even with moderate energy intake. In this context, GLP-1/GIP pharmacotherapy, while effective in initiating weight loss, may not fully counteract mechanisms that may promote weight regain if not accompanied by strategies to preserve muscle mass and dietary quality.

A growing number of authors emphasise that pharmacological appetite suppression is not equivalent to fully restoring physiological satiety mechanisms or improving micronutrient status [11,33]. The absence of adequate nutritional support during treatment may result in substantial weight loss accompanied by compromised body composition and metabolic reserves. In the post-treatment period, reduced energy expenditure, muscle loss, and potential micronutrient deficiencies may contribute to an environment conducive to weight regain and deterioration of metabolic function [9,10,14].

From a clinical perspective, this highlights the importance of treating GLP-1/GIP therapy not merely as a pharmacological intervention but as part of an integrated care model that includes monitoring protein intake, assessing body composition, and, where appropriate, periodic evaluation of selected micronutrients.

## 4. The Gut Microbiota as a Modulator of Satiety

Both obesity and prolonged caloric restriction are associated with reduced diversity of the gut microbiota and diminished production of short-chain fatty acids (SCFAs), particularly propionate and butyrate, which play important roles in regulating host metabolism and satiety signalling [13,14,21]. SCFAs interact with G-protein-coupled receptors (GPR41/FFAR3 and GPR43/FFAR2), contributing to the modulation of gut hormone secretion, including GLP-1 and PYY, and influencing inflammatory processes and insulin sensitivity [14,21,34].

These data derive from both preclinical studies and human intervention trials; however, their interpretation in the context of long-term weight control remains complex. SCFAs may enhance the secretion of satiety hormones and modulate vagal nerve activity, but this effect depends on the availability of fermentable substrates and microbiota composition [14,34].

These mechanisms should therefore be interpreted as biologically plausible but not yet established as causal drivers of post-pharmacotherapy weight regain in humans.

In the context of GLP-1/GIP pharmacotherapy, it is important to emphasise that pharmacological appetite suppression does not necessarily result in restoration of functional gut microbiota. Reduced energy intake, dietary simplification, and limited diversity of fermentable substrates may be associated with unfavourable changes in microbiota composition if not concurrently addressed through nutritional interventions.

A lack of improvement in microbiota structure and activity has been proposed as a biologically plausible contributor to impaired endogenous satiety signalling and increased susceptibility to weight regain; however, this has not yet been directly demonstrated after GLP-1/GIP discontinuation.

## 5. The Importance of Micronutrient Support

Following pharmacologically induced weight loss, ensuring adequate intake of selected micronutrients may become particularly important, as deficiencies may adversely affect metabolic and neurological functions and the body’s ability to adapt to a reduced body weight. Under conditions of prolonged energy restriction, reduced food volume, and selective limitation of certain food groups (e.g., fats and animal products), the risk of deficiencies in vitamin D, vitamin B12, iron, zinc, and magnesium may increase, especially without concurrent dietary planning and monitoring of nutritional status [11,28].

Currently, there are no established guidelines regarding routine micronutrient supplementation during GLP-1/GIP pharmacotherapy. However, given the reduction in energy intake observed in many patients and the potential risk of reduced dietary quality and suboptimal nutrient intake, this may represent an important gap in clinical practice that warrants further investigation. In clinical practice, it may be reasonable to consider micronutrient support as an element of secondary prevention during active pharmacotherapy and in the tapering phase.

This approach may be informed by evidence from clinical settings involving substantial weight loss and dietary restriction, such as bariatric surgery, where a high prevalence of micronutrient deficiencies has been consistently observed and routine monitoring and supplementation constitute standard components of care [35]. However, unlike bariatric surgery, GLP-1/GIP pharmacotherapy does not alter intestinal anatomy; therefore, the mechanisms and magnitude of deficiency risk are likely to differ substantially.

Weight reduction is not necessarily synonymous with improved nutritional quality: in some patients, energy restriction may be accompanied by a decline in dietary nutrient density, and the development of subclinical deficiencies may remain unrecognised until symptoms develop (fatigue, reduced exercise tolerance, cognitive impairment, mood disturbances), potentially hindering physical activity and the maintenance of treatment outcomes [11,35].

### 5.1. Fat-Soluble Vitamins, Dietary Fat, and Bioavailability

Vitamins A, D, E, and K require the presence of fat in the intestinal lumen and proper micellization for effective absorption; therefore, long-term maintenance of very low-fat diets may limit their bioavailability [28]. For this reason, it may be reasonable to maintain a minimum dietary fat intake and to include sources of dietary fat, such as plant-based oils (e.g., olive oil, rapeseed oil, oils rich in ALA), particularly in patients who adhere to low-fat diets due to gastrointestinal symptoms [28,36].

### 5.2. Vitamin D

Vitamin D plays a role not only in calcium-phosphate homeostasis but also in the modulation of the inflammatory response, muscle function, and insulin sensitivity. Vitamin D deficiency is common among individuals with obesity and may persist after weight loss, particularly in the context of limited dietary fat intake and low UV exposure [35,37]. Vitamin D supplementation may be tailored to baseline 25(OH)D levels and clinical risk, according to local recommendations [37]. Depending on the clinical context, assessment of vitamin K status may also be considered (particularly when supplementing vitamin D), although current data do not support universal “D3 + K2” supplementation strategies for all patients after pharmacotherapy.

### 5.3. Vitamin B12, Folates, Homocysteine

Vitamin B12 is essential for neurological and haematological function, and its intake may be suboptimal with low consumption of animal products and under conditions of reduced energy intake. In some patients (particularly those >50 years of age), the risk of B12 deficiency may increase due to impaired intrinsic factor-dependent absorption [38]. In cardiovascular and neurological contexts, it should be noted that homocysteine metabolism depends on B12, B6, and folates; correction of deficiencies may be relevant in selected patient groups, although routine empirical (“blind”) supplementation is not justified without clinical and/or laboratory assessment [38].

### 5.4. Magnesium

Magnesium plays a role in the regulation of glucose metabolism, muscle function, and nerve conduction. Magnesium deficiency is associated with poorer glycaemic control and may exacerbate fatigue and reduce exercise tolerance, potentially limiting the ability to engage in physical activity that is important for weight maintenance [39]. In patients with symptoms suggestive of deficiency or those on low-fibre/low-whole-grain diets, assessment of magnesium intake and possible supplementation may be considered.

### 5.5. Omega-3 Fatty Acids and Inflammation

Omega-3 fatty acids (EPA/DHA) have been shown to exert anti-inflammatory effects and may influence metabolic parameters, although the magnitude of clinical effects depends on dose, endpoints, and population [40]. In the context of obesity and inflammation, omega-3s may represent a supportive component of the overall metabolic strategy but do not replace foundational interventions such as protein intake, diet quality, and physical activity.

In summary, micronutrient support may be considered an element of integrated care for patients treated with GLP-1/GIP agents and may include risk assessment for deficiencies, prioritisation of likely deficiencies, and monitoring (clinical and, when justified, laboratory), particularly during the tapering phase of pharmacotherapy [11,35,37].

## 6. Clinical Implications

Available clinical and pathophysiological data suggest that obesity pharmacotherapy with GLP-1/GIP agonists, when applied without a concurrent nutritional and metabolic strategy, may have limited durability of effects. Intervention studies consistently demonstrate that treatment discontinuation is associated with weight regain in a substantial proportion of patients, likely reflecting biological adaptations that favour weight regain.

In this conceptual framework, this process is thought to involve persistent metabolic adaptations, disturbed hunger–satiety signalling, and incomplete restoration of fat-free mass. In this conceptualisation, pharmacotherapy primarily functions to modulate appetite and energy balance, while its cessation may unmask compensatory mechanisms favouring weight regain.

From a clinical practice perspective, this highlights the importance of implementing a systemic treatment model in which pharmacotherapy constitutes one element of a broader therapeutic strategy. Building stable, endogenous satiety after therapy reduction or cessation is likely to require:

- Preservation of fat-free mass (adequate protein intake, resistance training);
- Stabilisation of postprandial glycaemia (low glycaemic load) [41,42];
- Maintenance of dietary nutrient density;
- Support of the gut–liver–brain axis through adequate intake of fermentable fibre [9,10,11,14,21,22].

Failure to integrate these elements may result in a state in which, after drug discontinuation, the patient functions under conditions of reduced energy expenditure and heightened hunger, increasing susceptibility to weight regain [5,9,10,23,24].

The gut microbiota remains an important, though still insufficiently understood, element of metabolic stabilisation. Mechanistic data suggest that reduced SCFA production may weaken satiety signalling and contribute to persistent low-grade inflammation, although the clinical relevance of this mechanism after GLP-1/GIP discontinuation remains to be established [13,14,21,34].

However, the current state of knowledge does not permit treating probiotics or prebiotics as independent strategies for preventing weight regain. Microbiota-targeted interventions are best considered as adjunctive components of a comprehensive approach encompassing diet quality, fermentable fibre, and physical activity [43,44,45,46].

From the clinician’s standpoint, individualisation of post-pharmacotherapy strategies is particularly important, taking into account metabolic phenotype, degree of muscle mass loss, dietary quality, and risk of micronutrient deficiencies. The absence of such an approach may lead to weight regain being interpreted as a “treatment failure”, whereas it more accurately reflects the chronic, biologically determined nature of obesity.

Table 1 summarises biological mechanisms that may contribute to weight regain after GLP-1/GIP therapy cessation, beyond behavioural factors. Understanding these processes is crucial for planning individualised post-pharmacotherapy strategies.

## 7. Conclusions

Obesity is a systemic disease involving complex endocrine, metabolic, inflammatory, and neurohormonal disturbances; therefore, its treatment should not be limited to short-term weight reduction. Pharmacotherapy with GLP-1/GIP agonists represents a significant therapeutic advance, but available data indicate that after its cessation, sustained restoration of the mechanisms regulating satiety and energy balance often remains incomplete, and weight regain is commonly observed and is likely influenced by persistent biological adaptations.

Long-term weight stabilisation may require a gradual transition from pharmacologically induced satiety to satiety supported by diet quality, stable glycaemia, preserved body composition, and the gut–liver–brain axis. This transition should be regarded as a conceptual framework and forward-looking hypothesis requiring validation in prospective studies.

This process is supported by preserving fat-free mass, ensuring adequate protein intake, preventing micronutrient deficiencies, and supporting functional activity of the gut microbiota.

GLP-1/GIP pharmacotherapy should therefore be viewed as a component of an interdisciplinary model of obesity treatment, in which nutritional and metabolic interventions are key components of maintaining therapeutic outcomes. Further clinical research is needed to determine which post-pharmacotherapy strategies most effectively support sustained weight stabilisation and in which patients’ therapy may be safely reduced or discontinued without significant risk of relapse.

## Figures and Tables

**Table 1:** Biological drivers of weight regain after GLP-1/GIP pharmacotherapy withdrawal.

Mechanism	Physiological Change	Clinical Consequence
Adaptive thermogenesis	↓ Resting energy expenditure beyond predicted values	Positive energy balance at previously weight-stable intake
Hormonal adaptation	↑ Ghrelin, ↓ leptin signalling	Increased hunger and reduced satiety
Lean mass loss	↓ Fat-free mass	Lower metabolic rate and reduced metabolic flexibility
Microbiota dysregulation	↓ SCFA production	Impaired gut-derived satiety signalling
Residual hepatic insulin resistance	Potentially altered hepatic fuel sensing	Possible contribution to impaired metabolic regulation

## Data Availability

No new data were created or analysed in this study.
